# Supplementary figures and images for: Integrative Multi-Omics Analyses Reveal the Global Regulation Network of the Microalga Nannochloropsis oceanica Under Nitrogen Stress Adaptation
Source: Biology (Basel). 2025 Nov 15;14(11):1599. doi: 10.3390/biology14111599 (PMC12649847; doi:10.3390/biology14111599)

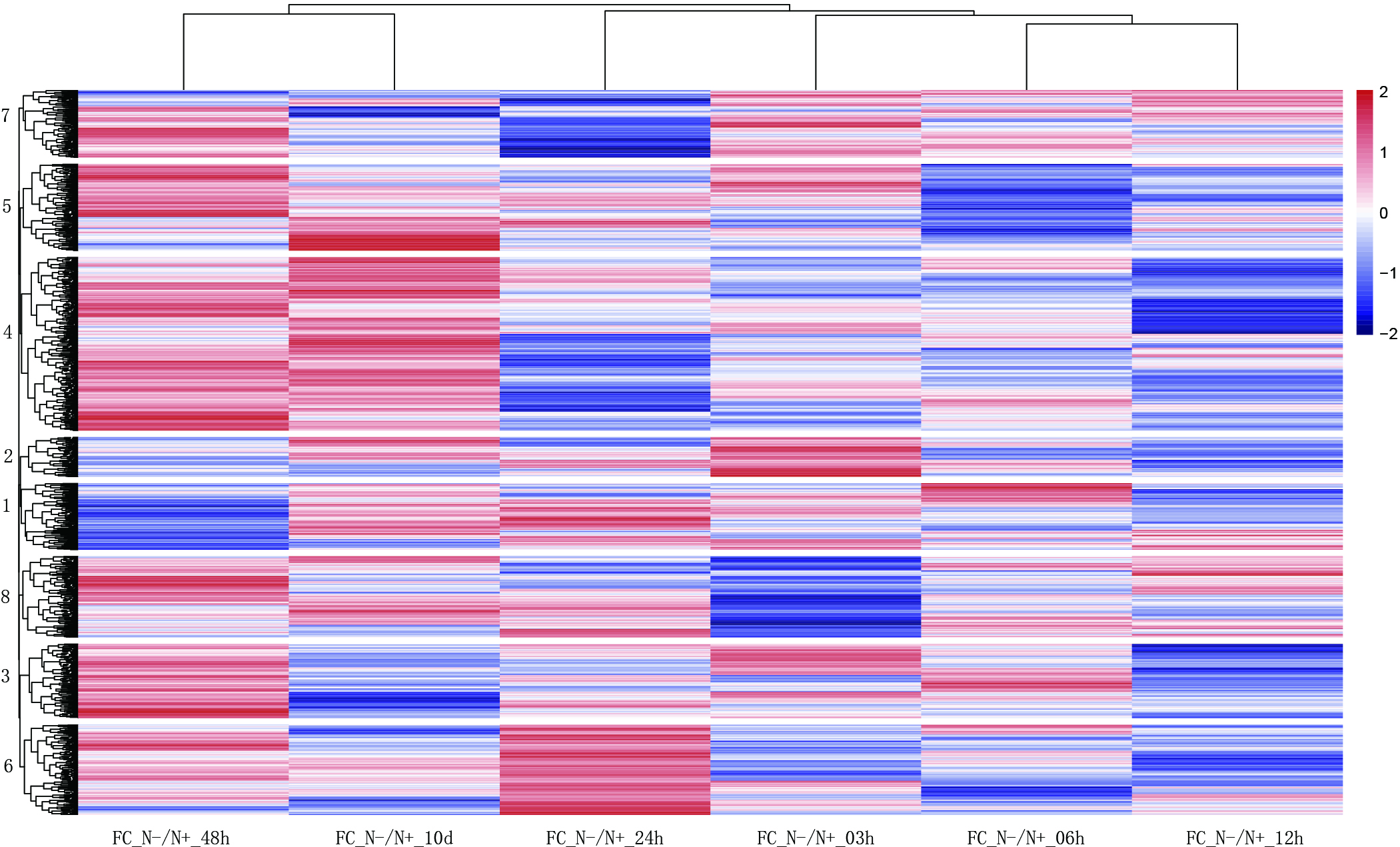

Supplement: Supplementary file 1 [file biology-14-01599-s001.zip › biology-3927214-supplementary.jpg]
